# Simultaneous activation of innate and adaptive immunity participates in the development of renal injury in a model of heavy proteinuria

**DOI:** 10.1042/BSR20180762

**Published:** 2018-07-13

**Authors:** Viviane Dias Faustino, Simone Costa Alarcon Arias, Victor Ferreira Ávila, Orestes Foresto-Neto, Fernanda Florencia Fregnan Zambom, Flavia Gomes Machado, Luciene Machado dos Reis, Denise Maria Avancini Costa Malheiros, Rildo Aparecido Volpini, Niels Olsen Saraiva Camara, Roberto Zatz, Clarice Kazue Fujihara

**Affiliations:** 1 Renal Division, Department of Clinical Medicine, Faculty of Medicine, University of São Paulo, São Paulo, Brazil; 2 Laboratory of Transplantation Immunobiology, Institute of Biomedical Sciences, University of São Paulo, São Paulo, Brazil

**Keywords:** adaptive immunity, chronic kidney disease, NLRP3 inflammasome, NF-κB system, proteinuria

## Abstract

Protein overload of proximal tubular cells (PTCs) can promote interstitial injury by unclear mechanisms that may involve activation of innate immunity. We investigated whether prolonged exposure of tubular cells to high protein concentrations stimulates innate immunity, triggering progressive interstitial inflammation and renal injury, and whether specific inhibition of innate or adaptive immunity would provide renoprotection in an established model of massive proteinuria, adriamycin nephropathy (ADR). Adult male Munich–Wistar rats received a single dose of ADR (5 mg/kg, iv), being followed for 2, 4, or 20 weeks. Massive albuminuria was associated with early activation of both the NF-κB and NLRP3 innate immunity pathways, whose intensity correlated strongly with the density of lymphocyte infiltration. In addition, ADR rats exhibited clear signs of renal oxidative stress. Twenty weeks after ADR administration, marked interstitial fibrosis, glomerulosclerosis, and renal functional loss were observed. Administration of mycophenolate mofetil (MMF), 10 mg/kg/day, prevented activation of both innate and adaptive immunity, as well as renal oxidative stress and renal fibrosis. Moreover, MMF treatment was associated with shifting of M from the M1 to the M2 phenotype. In cultivated NRK52-E cells, excess albumin increased the protein content of Toll-like receptor (TLR) 4 (TLR4), NLRP3, MCP-1, IL6, IL-1β, Caspase-1, α-actin, and collagen-1. Silencing of *TLR4* and/or *NLRP3* mRNA abrogated this proinflammatory/profibrotic behavior. Simultaneous activation of innate and adaptive immunity may be key to the development of renal injury in heavy proteinuric disease. Inhibition of specific components of innate and/or adaptive immunity may be the basis for future strategies to prevent chronic kidney disease (CKD) in this setting.

## Introduction

Chronic kidney disease (CKD) is a growing cause of deaths and morbidity worldwide. Measures to delay the progression of CKD to end-stage renal disease are currently limited, and new strategies are needed [1,2].

Tubulointerstitial inflammation plays a central role in the pathogenesis of CKD, but the exact mechanisms behind these effects remain largely unknown [3–5]. A number of *in vivo* and *in vitro* studies have provided evidence that not only proteinuria is a hallmark of kidney disease, but also that excessive protein reabsorption is a pathogenic factor involved in the development of CKD, leading proximal tubular cells (PTC) to produce proinflammatory and profibrotic mediators [6–8]. However, the mechanisms underlying this effect are unclear.

We have recently shown that nearly simultaneous activation of both innate and adaptive immunity occurs soon after 5/6 renal ablation (Nx) [9], persisting throughout the evolution of this CKD model. Other recent clinical and experimental observations have also suggested that innate immunity is activated in the setting of kidney disease [10–14]. Accordingly, treatment with anti-inflammatory drugs, such as mycophenolate mofetil (MMF), and even non-steroidal anti-inflammatory drugs, has been shown to be renoprotective in widely used CKD models, such as Nx, chronic NO inhibition and streptozotocin diabetes [15–19]. However, the reasons why inflammation develops in renal tissue even when CKD is not initiated by an immune dysfunction or the presence of foreign antigens are unclear.

Several innate immune components are activated in CKD [20–25], such as the NF-κB system, Toll-like receptors (TLRs 1–9), NOD receptors and the NLRP3 inflammasome [26–29], a cytoplasmic multiprotein complex containing the NOD2 receptor and the ASC adapter, which is required for the production of caspase-1 and the consequent release of the potent proinflammatory interleukins IL-1β and IL-18 [10,25,28,29]. Activation of innate immunity pathways in CKD may represent an important link between non-specific insults, for example tubular exposure to high protein concentrations, and the development of renal fibrosis and glomerulosclerosis [30–34].

In the present study, we investigated the hypothesis that prolonged exposure of tubular cells to high protein concentrations stimulates innate immunity, thus activating the inflammatory cascade and the development of CKD, and that inhibition of either innate or adaptive immunity promotes renoprotection in adriamycin nephropathy (ADR), a model of heavily proteinuric kidney disease.

## Materials and methods

### Animal studies

Adult male Munich–Wistar rats, weighing 230–250 g, were obtained from a local facility. The experimental procedures were approved by the local Research Ethics Committee (CAPPesq, process number 108/15) and developed in strict conformity with Institutional Guidelines and International Standards for Manipulation and Care of Laboratory Animals. Rats were given free access to tap water, fed regular chow (Nuvital Labs, Curitiba, Brazil), and kept at 23 ± 1°C and 60 ± 5% relative air humidity under an artificial 12:12-h light/dark cycle. For ADR administration, 35 rats were anesthetized with ketamine (2 mg/kg im) and xylazine (0.4 mg/kg im), and received a single iv injection of 5 mg/kg doxorubicin hydrochloride (Sigma) diluted in 0.9% saline. Rats were followed for 2, 4, or 20 weeks. Control rats (C group; *n*=18) received no treatment. In a second protocol, we studied 32 rats receiving ADR only and 30 rats receiving ADR and MMF (Roche Laboratories, Nutley, NJ), dissolved in 5% DMSO, then in olive oil [18], and given by gavage at 10 mg/kg/day. At the end of each protocol, tail-cuff pressure (TCP) was determined with an optoelectronic automated device (BP 2000 Blood Pressure Analysis System, Visitech Systems, EUA) after rats had been preconditioned to the procedure [35], and urinary albumin excretion (ALB) was assessed by radial immunodiffusion. On the following day, the animals were anesthetized with ketamine (50 mg/kg im) and xylazine (10 mg/kg im). Blood samples were taken from the abdominal aorta for biochemical analyses, and the right kidney was retrogradely perfused *in situ* with saline through the abdominal aorta to remove blood from renal vessels and after excised, rapidly frozen at −80°C for protein assessment or isolation of nuclei. The left kidney was then perfusion fixed with Duboscq-Brazil solution and, subsequently, fixed in buffered 10% formaldehyde solution, and embedded in paraffin by conventional techniques. Histomorphometric and immunohistochemical analyses of the renal tissue were performed in 4-mm thick sections.

### Biochemical and enzymatic analyses

Serum creatinine (SCr) and triglycerides (TGs) were determined using a commercial kit (Labtest Diagnostica, São Paulo, Brazil). Plasma K^+^ concentration was determined with a conventional ion-selective electrode.

### Histomorphometry

Morphometric evaluations were always performed in a blinded manner by a single observer. The frequency of glomeruli with sclerotic lesions was determined in PAS-stained sections as described previously [36].

### Immunohistochemical analysis

Sections were mounted on 2% silane-coated glass slides. The following primary antibodies were employed: monoclonal mouse anti-rat ED-1 (Serotec, Oxford, U.K.); polyclonal rabbit anti-mannose receptor CD206 (Abcam, Cambridge, U.K.) for M2 macrophages; monoclonal mouse anti-human CD3 (Dako, Glostrup, Denmark); polyclonal rabbit anti-collagen I (Abcam, Cambridge, U.K.); monoclonal mouse anti-α-SMA (Sigma, St. Louis, U.S.A.); and polyclonal rabbit anti-NLRP3 (Sigma, St. Louis, U.S.A.). Procedures for detection of CD3, ED-1, and NLRP3, collagen-1, and α-SMA have been detailed elsewhere [64]. For M2 macrophages detection (CD206), sections were pretreated with 30% hydrogen peroxide in methanol and pre incubated with serum-free protein block (Dako, Glostrup, Denmark). The primary antibody was diluted at 1:10000 in 1% BSA. After rinsing with TBS, sections were incubated with HRP-labeled polymer conjugated with secondary antibodies (Dako, Glostrup, Denmark), then with DAB substrate-chromogen solution (Dako, Glostrup, Denmark) for development. For the estimation of cortical interstitial renal density of ED-1, M2, CD3, and NLRP3, the number of positive cells/mm^2^ was evaluated in a blinded manner at 400× magnification. The percentage of cortical interstitial area occupied by collagen I and α-SMA was estimated by a point-counting technique. For each section, 50 microscopic fields (~1.6 mm^2^) were examined.

### Total protein extraction and isolation of nuclei

Renal proteins were extracted using lysis buffer (Thermo Scientific, Rockford, U.S.A.) with protease and phosphatase inhibitors (Roche, Mannheim, Germany). Protein concentration was determined by the BCA method. For isolation of nuclei, kidneys were homogenized on ice in lysis buffer and centrifuged at 1000 ***g*** for 10 min at 4°C to obtain a crude nuclear pellet. The pellet was further reconstituted in high-sucrose Laemmli buffer and centrifuged at 1500 ***g*** for 10 min at 4°C to obtain a pure nuclear suspension.

### Cell culture studies

Immortalized NRK-52E PTCs (American Type Culture Collection), were maintained in Dulbecco’s modified Eagle’s medium (DMEM)/F12 (Invitrogen, Carlsbad, CA) containing 10% FBS and 1% penicillin–streptomycin, and incubated at 37°C and 5% CO_2_. After confluence, cells were trypsinized, fresh medium was substituted every 2 days, and cells were subcultured every 7 days after seeding. Cells were then exposed to BSA (10 mg/ml) (Sigma–Aldrich, Schnelldorf, Germany) for 24 h and then processed as described later. Transfection reagents and siRNA for Nlrp3 and/or TLR4, were obtained from Invitrogen (RNAiMax reagent and Stealth RNAi™). The non-targetting scramble-sequence siRNA was used as a negative control. Cultured PTCs were transfected according to the manufacturer’s protocol (Invitrogen, Carlsbad, U.S.A.). Efficiency of transfection reagents was assessed by fluorescent immunocytochemistry staining analysis for TLR4 and NLRP3. Cell viability/proliferation was assessed using the MTT cell proliferation assay (Thermo Fisher Scientific, Darmstadt, Germany).

### Fluorescence immunocytochemistry

Cells were fixed with 4% paraformaldehyde. After serum blocking for 30 min, cells were incubated with the primary antibody to polyclonal rabbit anti-NLRP3 (Sigma, St. Louis, U.S.A.); polyclonal rabbit anti-TLR4 (Santa Cruz Biotechnology, Dallas, U.S.A.); polyclonal rabbit anti-IL-1β (Santa Cruz Biotechnology, Dallas, U.S.A.); monoclonal mouse IL-6 (Abcam, Cambridge, United Kingdom); or monoclonal mouse anti-α-SMA (Sigma, St. Louis, U.S.A.), then with rhodamine- or FITC-conjugated secondary antibody (Life Technologies, Carlsbad, EUA). Finally, cells were counterstained with DAPI and mounted on glass slides. PTC profiles containing fluorescent material were expressed as a percentage of the total number of nuclei. Multiple images were taken per group and the mean fluorescence intensity (MFI) of the stainings was quantitated using ImageJ software and calculated in relation to the number of nuclei per field.

### Western blot assays

Proteins from cells and kidney tissue were extracted using RIPA lysis buffer and protein concentration was determined using a Bradford assay. One hundred micrograms of total proteins were mixed with 2× Laemmli loading buffer and denatured at 96°C for 5 min. For nuclear fraction analysis, the preconditioned samples were not denatured. Protein separation was performed by SDS/PAGE. Proteins were transferred on to a nitrocellulose membrane (Amersham Biosciences, Little Chalfont, U.K.) and were incubated with 5% non-fat milk or 5% BSA in TBS for 1 h at room temperature to block non-specific binding. The membrane was then incubated overnight at 4°C with primary antibodies for: β-actin, 1:5000 (Sigma–Aldrich, St. Louis, U.S.A.); collagen-1, 1:500 (Abcam, Cambridge, United Kingdom); TLR4, 1:250 (Santa Cruz Biotechnology, Dallas, U.S.A.), caspase-1, 1:1000 (Santa Cruz Biotechnology, Santa Cruz, U.S.A.); heme oxygenase 1 (HO-1), 1:500 (Abcam, Cambridge, United Kingdom); superoxide dismutase 2 (SOD2), 1:10000 (Cayman, Michigan, U.S.A.); p-NF-κB p65 component, 1:100 (Cell Signaling, Danvers, U.S.A.); histone H2B, 1:1500 (Abcam, Cambridge, United Kingdom); or IL-6 (Abcam, Cambridge, United Kingdom). After rinsing with TBS Tween 20 (TBST) buffer, membranes were incubated with secondary antibodies labeled with HRP. Immunostained bands were detected using a chemiluminescence kit (Thermo Scientific, Rockford, U.S.A.), and were further analyzed by densitometry with a gel documentation system and the Ubisoft-UvibandMax software (Uvitec Cambridge, Cambridge, United Kingdom).

### ELISA

Protein lysates obtained from frozen samples of renal cortex were used for ELISA analysis of the content of NGAL, IL-1β and IL-10. The concentration of NGAL was determined in the urine, using a rat NGAL ELISA kit (Bioporto, Copenhagen, Denmark). Total IL-1β and IL-10 were measured in homogenized kidney tissues and cell supernatant MCP-1 was quantitated using a commercial kit (R&D Systems, Minneapolis, U.S.A.). All analyses were performed following the manufacturer’s instructions.

### Statistical analysis

Results were expressed as means ± S.E.M. Statistical differences amongst groups were assessed by one-way ANOVA (with Newman–Keuls post-test). Correlations were determined by calculating the Pearson’s coefficient. Differences were considered significant at *P*<0.05. All calculations were performed using GraphPad Prism 4.0 software.

## Results

ADR caused massive albuminuria, hypoalbuminemia, weight loss, anemia, and hypertriglyceridemia (Table 1). These changes were accompanied by hypertension, renal hypertrophy, glomerulosclerosis, and interstitial fibrosis. All these abnormalities reached a maximum of 20 weeks after ADR administration, when SCr had also become elevated (Supplementary Figure S1A–F). Likewise, a significant increase in urinary NGAL excretion was observed from as early as 2 weeks after ADR injection, along with a parallel increase in serum K^+^ (Supplementary Figure S2A,B).

**Table 1:** Body weight, serum albumin, hematocrit, and TGs 2, 4, and 20 weeks after adriamycin injection

	C	ADR_2W_	ADR_4W_	ADR_20W_
**BW, g**	314 ± 20	239 ± 7^1^	235 ± 7^1^	268 ± 22^1^
[**SALB**]**, mg/dl**	3.5 ± 0.2	1.4 ± 0.2^1^	1.3 ± 0.3^1^	1.3 ± 0.1^1^
**TG, mg/dl**	47 ± 8	202 ± 28^1^	715 ± 33^1,2^	335 ± 46^1,2,3^
**Ht, %**	50 ± 1	46 ± 1^1^	44 ± 1^1^	44 ± 1^1^

Abbreviations: BW, body weight; SALB, serum albumin; Ht, hematocrit in C, ADR_2W_, ADR_4W_, and ADR_20W_ groups. Results are expressed as means ± S.E.M. ^1^*P*<0.05 compared with C. ^2^*P*<0.05 compared with ADR_2W_. ^3^*P*<0.05 compared with ADR_4W_.

ADR administration was associated with early interstitial infiltration by lymphocytes and macrophages, along with an increase in the protein content of MCP-1 (Supplementary Figure S3A,B,C respectively).

Representative microphotographs of glomerulosclerosis (PAS); interstitial collagen-1 deposition; and infiltration by myofibroblasts, macrophages, and lymphocytes are given in Supplementary Figure S4.

**Figure 1:**
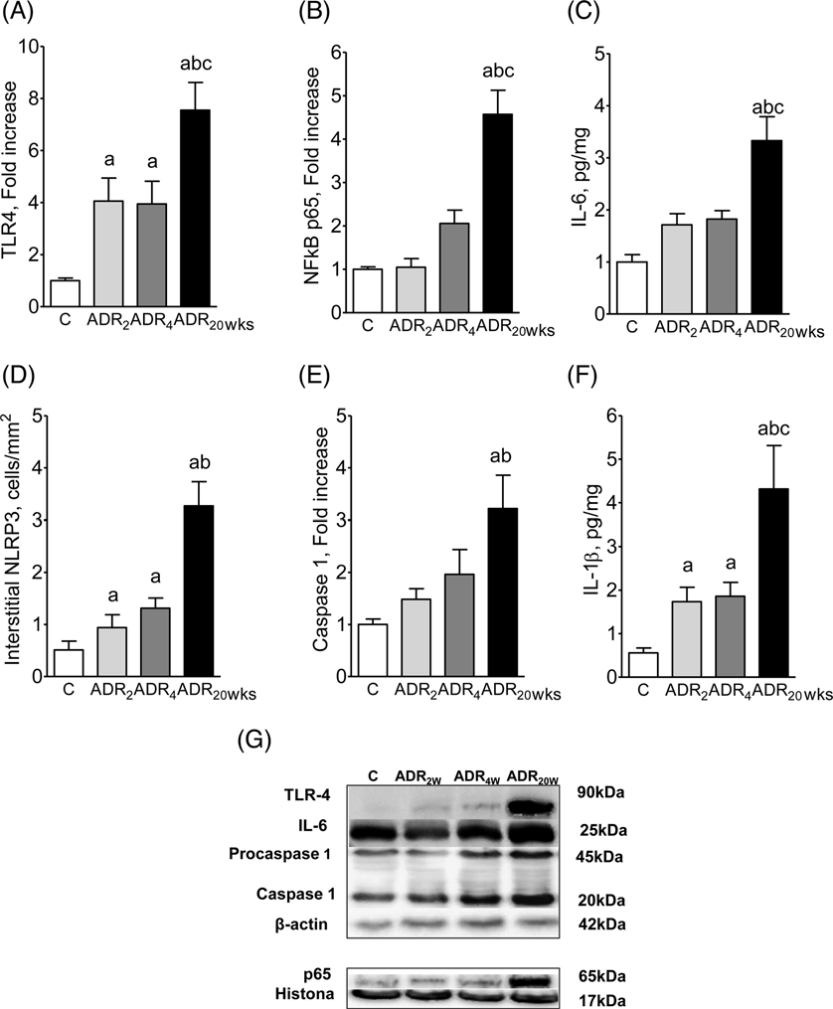
NLRP3 and NF-κB patwhays 2, 4 and 20 weeks after adriamycin injection Renal protein content of TLR4 (**A**), nuclear p65 (**B**), IL-6 (**C**), interstitial NLRP3+ cells (**D**), caspase-1 (**E**), and IL-1β 2 (**F**) 2, 4, and 20 weeks after ADR injection; representative Western blot images appear at the bottom (**G**). C *n*=9, ADR_2w_*n*=12, ADR_4w_*n*=12, ADR_20w_*n*=10. ANOVA ^a^*P*<0.05 compared with C; ^b^*P*<0.05 compared with ADR_2w_; ^c^*p*<0.05 compared with ADR_4w_.

The renal protein content of TLR4, nuclear p65, IL-6 (indicating activation of the NF-κB system), interstitial NLRP3 (cells/mm^2^), caspase-1, and IL-1β, shown in Figure 1A–G, followed a similar pattern of progressive increase, already significant at 2 weeks in the case of TLR4 and IL-1β.

**Figure 2:**
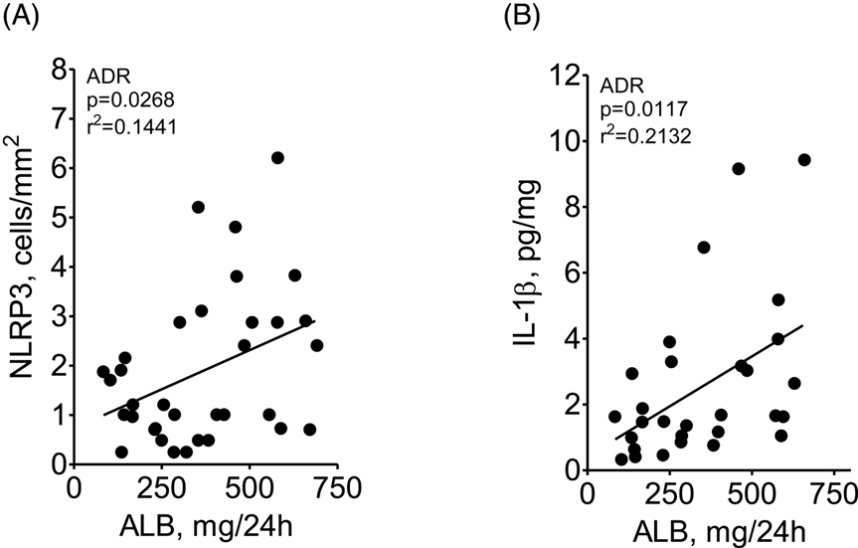
Linear correlation between albuminuria and renal NLRP3 or 1L-1β abundance Linear correlation (Pearson’s correlation coefficient) between the intensity of albuminuria and the abundance of NLRP3 (**A**) and IL-1β (**B**). Additional correlations between albuminuria and parameters of inflammation and innate immunity activation are shown in Supplementary data.

The intensity of albuminuria correlated significantly with the abundance of NLRP3 (cells/mm^2^) and IL-1β (pg/mg), as shown in Figure 2A,B. Additionally, albuminuria correlated with cortical content of α-SMA and collagen-1 (Supplementary Figure S5) and the density of renal lymphocyte infiltration correlated with content of NLRP3 and IL-1β (Supplementary Figure S6).

**Figure 3:**
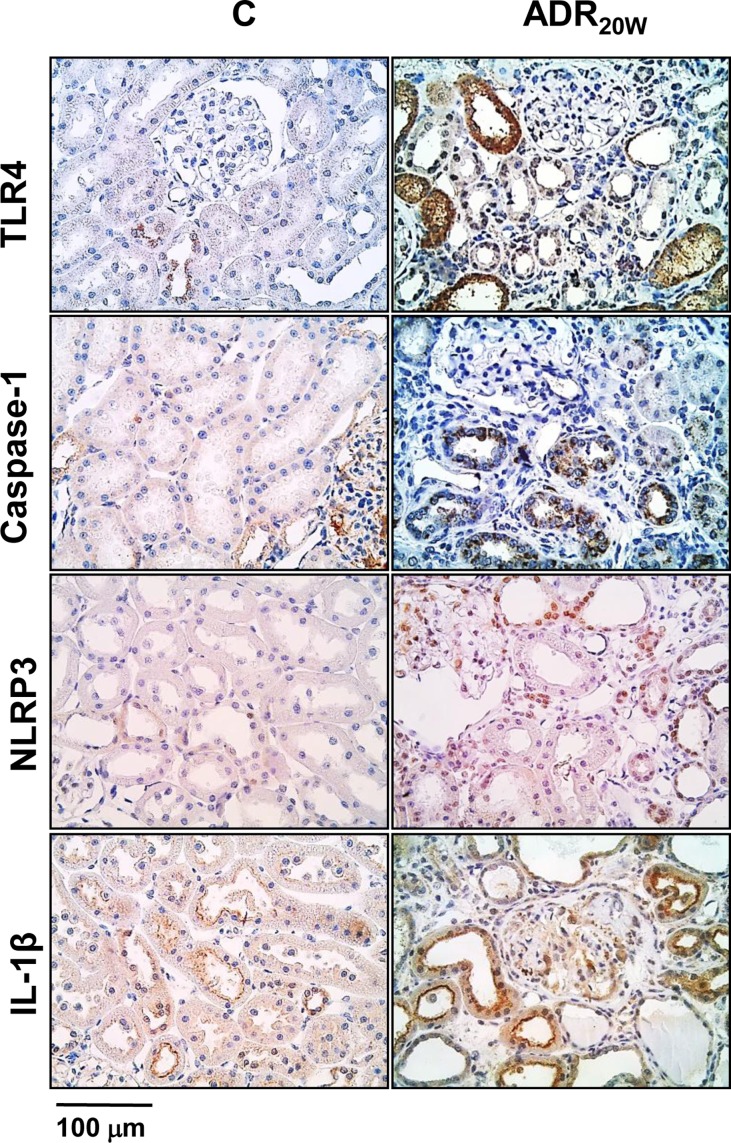
Representative microphotographs showing detection by immunohistochemistry of TLR4, Caspase-1, NLRP3, and IL-1β, the predominant staining is located in the tubular and interstitium (renal tissue)

In Figure 3, detection of TLR4, Caspase-1, NLRP3, and IL-1β in the renal tissue by immunohistochemistry is illustrated in representative microphotographs.

**Figure 4:**
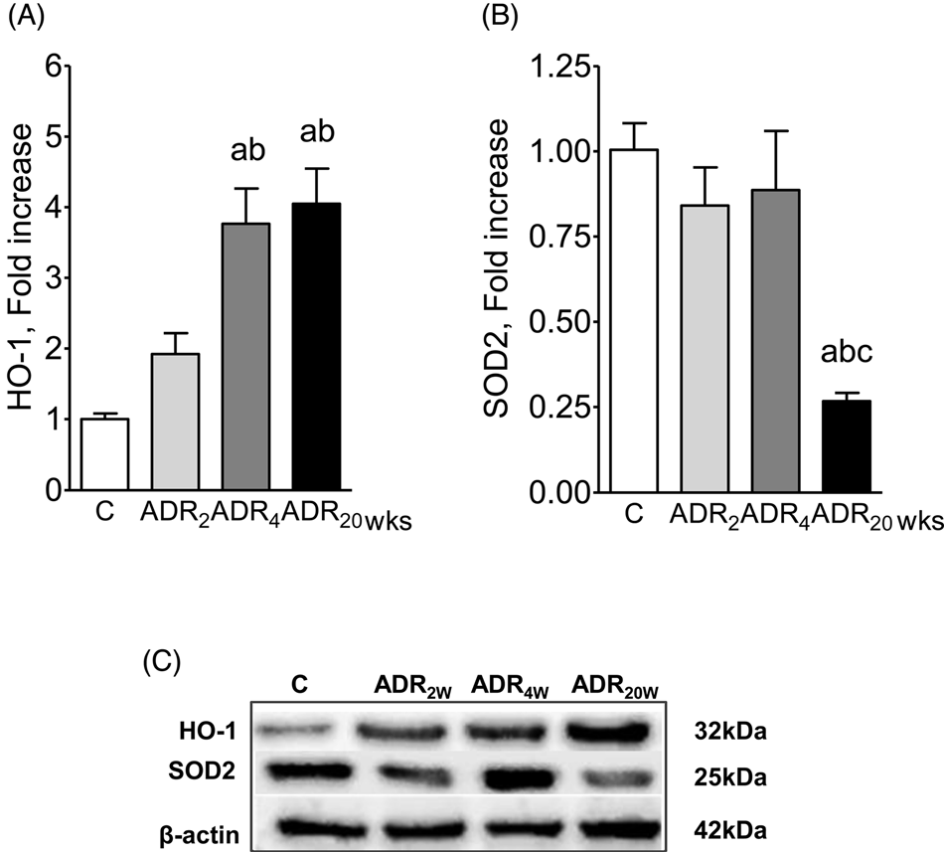
Markers of oxidative stress after adriamycin injection Renal abundance of HO-1 (**A**) and SOD2 (**B**) 2, 4, and 20 weeks after ADR injection. Representative Western blot images appear at the bottom (**C**). C *n*=9, ADR_2w_*n*=12, ADR_4w_*n*=12, ADR_20w_*n*=10. ANOVA ^a^*P*<0.05 compared with C; ^b^*P*<0.05 compared with ADR_2w_; ^c^*P*<0.05 compared with ADR_4w_.

Figure 4A shows progressive increase in the renal abundance of HO-1 after ADR injection. Figure 4B shows a significant fall in the renal content of SOD2 20 weeks after ADR injection. The corresponding Western blots are shown in Figure 4C. Together these findings indicate that progressive oxidative stress occurred in the kidneys of ADR-treated animals.

**Figure 5:**
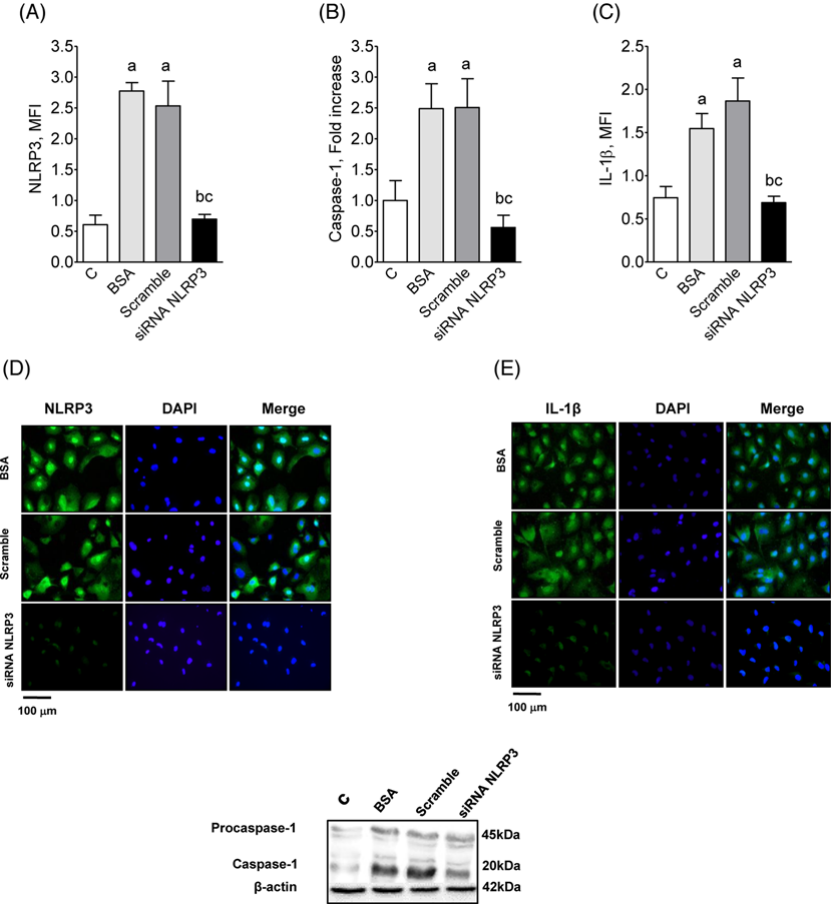
NLRP3, Casp-1 and IL-1β production by cultivated proximal tubular cells after exposure to BSA Production of NLRP3 (**A**), Caspase-1 (**B**), and IL-1β (**C**) by cultivated PTCs exposed to BSA and transfected with either scramble or siRNA for NLRP3. Illustrative immunofluorescence microphotographs of NLRP3 (**D**) and IL-1β (**E**) in these cells are also shown, while representative Western blot images appear at the bottom. ANOVA ^a^*P*<0.05 compared with C; ^b^*P*<0.05 compared with BSA; ^c^*P*<0.05 compared with scramble.

**Figure 6:**
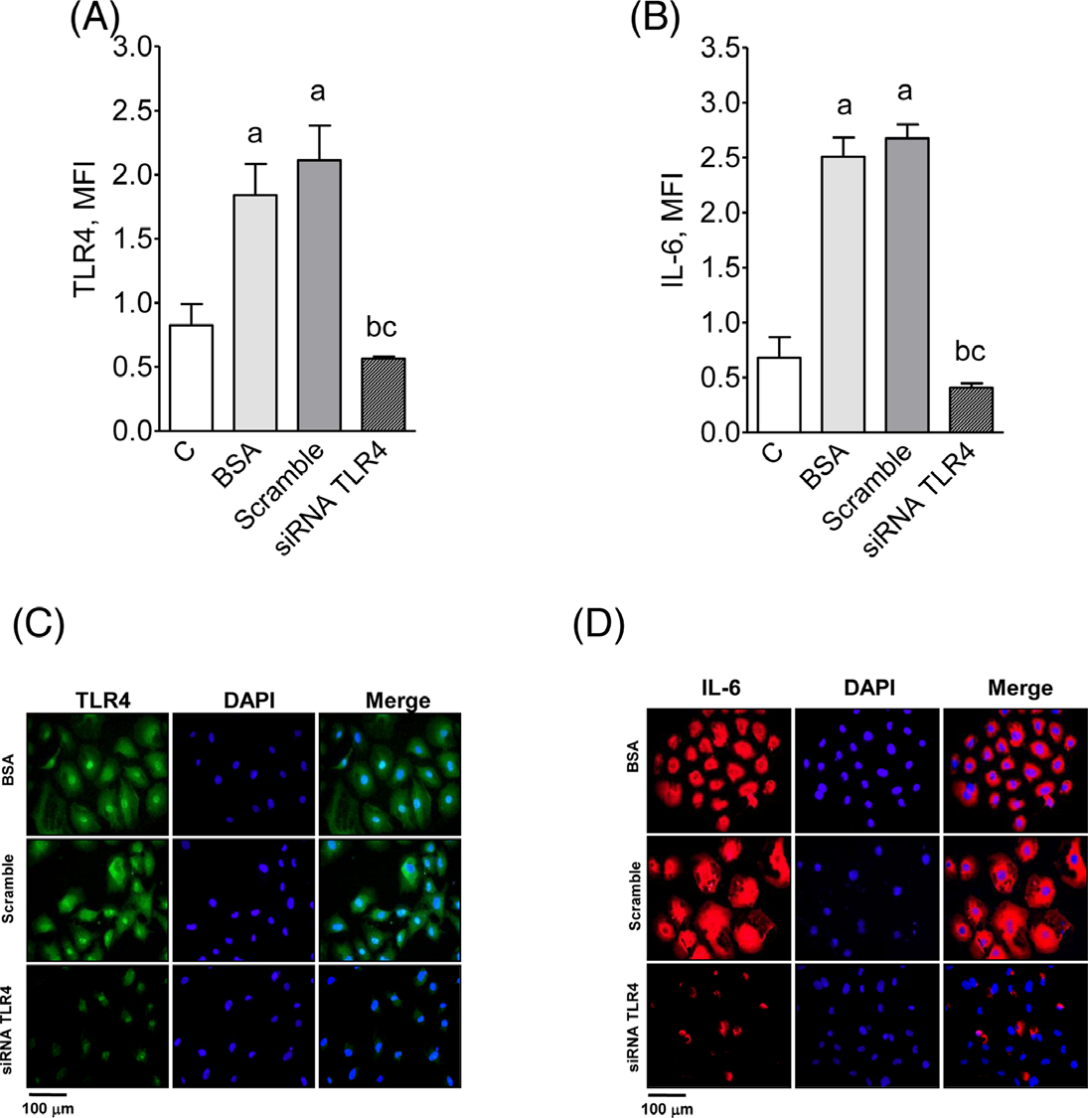
TLR4 and IL-6 production by cultivated proximal tubular cells after exposure to BSA Production of TLR4 (**A**) and IL-6 (**B**) by cultivated PTCs exposed to BSA and transfected with either scramble or siRNA for TLR-4. Illustrative immunofluorescence microphotographs of TLR-4 (**C**) and IL-6 (**D**) in these cells are also shown. ANOVA ^a^*P*<0.05 compared with C; ^b^*P*<0.05 compared with BSA; ^c^*P*<0.05 compared with scramble.

**Figure 7:**
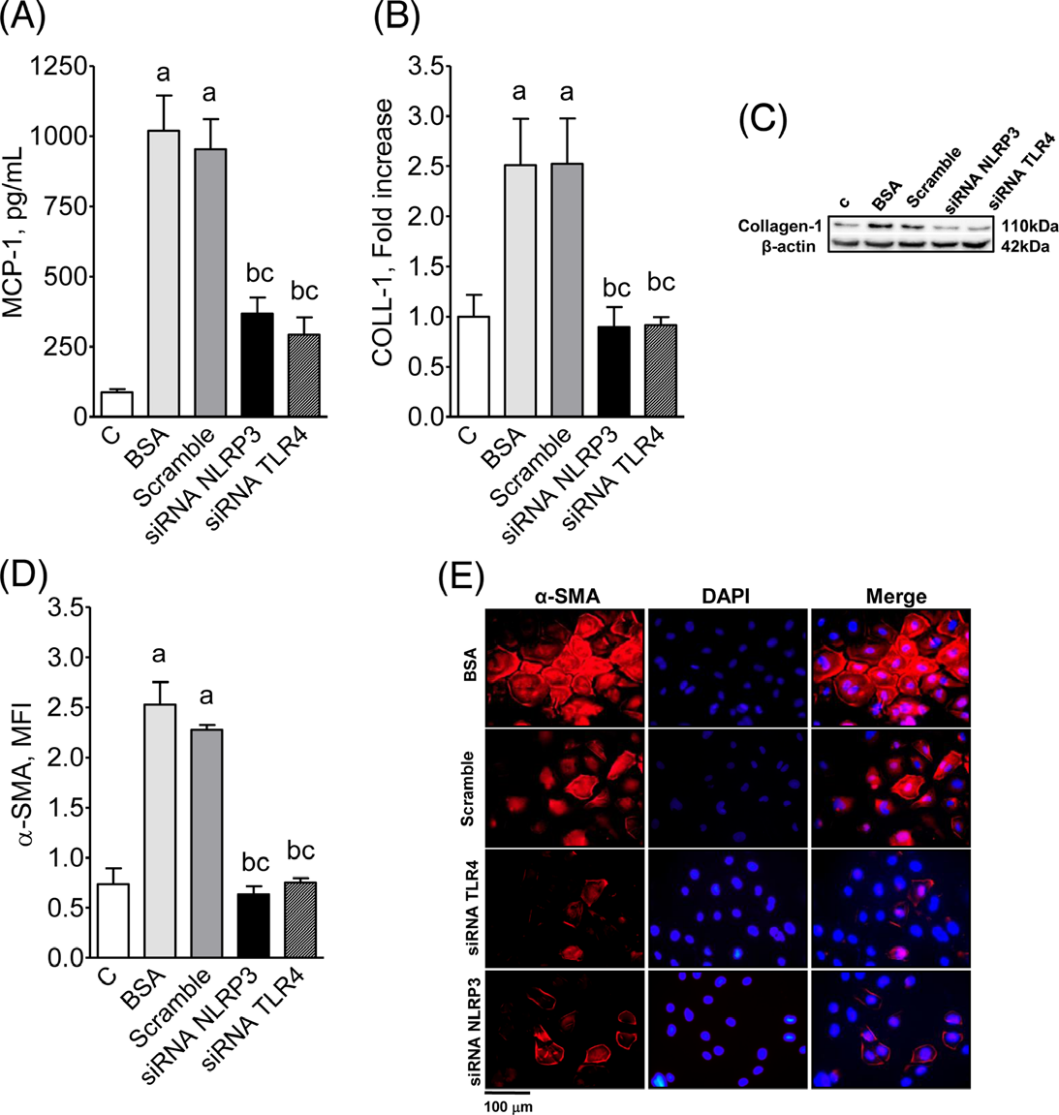
MCP-1, COLL-1 and α-SMA production by cultivated proximal tubular cells after exposure to BSA Production of MCP-1 (**A**), collagen-1 (**B**), and α-SMA (**D**) by cultivated PTCs exposed to BSA and transfected with scramble, siRNA for NLRP3, or siRNA for TLR-4. Illustrative immunofluorescence microphotographs of α-SMA (**E**) in these cells are also shown, while representative Western blot images appear in (**C**). ANOVA ^a^*P*<0.05 compared with C; ^b^*P*<0.05 compared with BSA; ^c^*P*<0.05 compared with scramble.

Consistent with *in vivo* observations, exposure of cultured murine PTCs to BSA (Figure 5A–E) augmented the production of NLRP3, Caspase-1, and IL-1β, indicating activation of the NLRP3 inflammasome pathway. In addition, the increased production of TLR4 and IL-6 (Figure 6) suggests that the NF-κB system was activated as well. The enhanced production of MCP-1, collagen-1, and α-SMA (Figure 7) indicates that PTCs exposed to BSA acquired a proinflammatory phenotype. Silencing of the *Tlr4* or *Nlrp3* genes prevented the excess production of each of these innate immunity components, abrogating the proinflammatory behavior of these cells (Figures 5–7). The proportion of viable cultivated cells, as assessed by the MTT test, was not different amongst the groups studied (Supplementary Figure S7).

**Figure 8:**
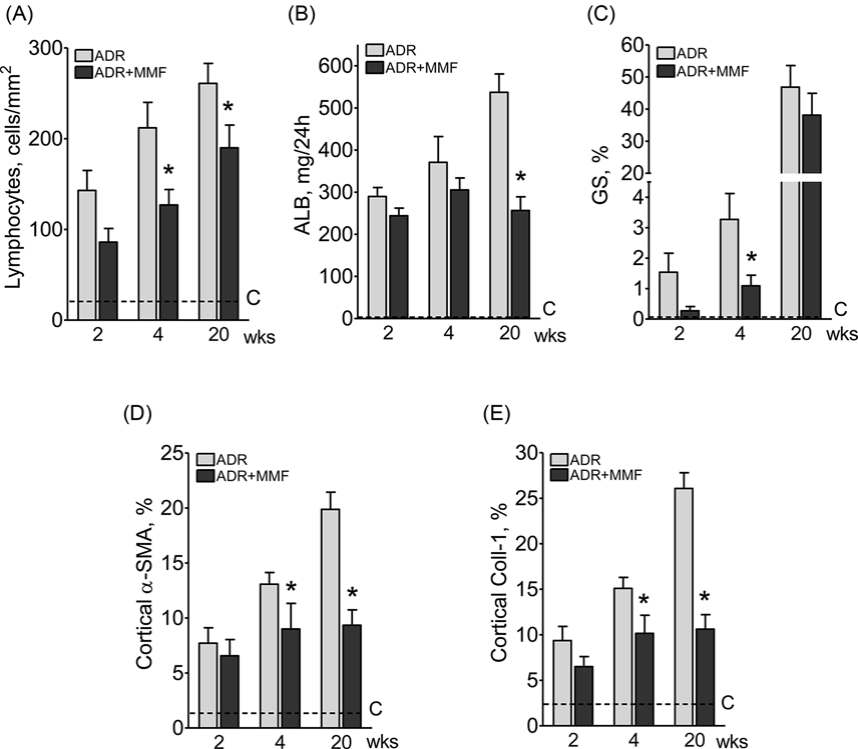
Effect of MMF treatment on renal injury and inflamation after adriamycin injection Effect of MMF treatment on renal T-lymphocyte (CD3-positive) infiltration (**A**), albuminuria (**B**), percent glomerulosclerosis (**C**), cortical α-SMA (**D**), and collagen-1 deposition (**E**) 2, 4, and 20 weeks after ADR injection, *n*=10 per group. Student’s ‘*t*’ test; *P<0.05 compared with ADR.

**Figure 9:**
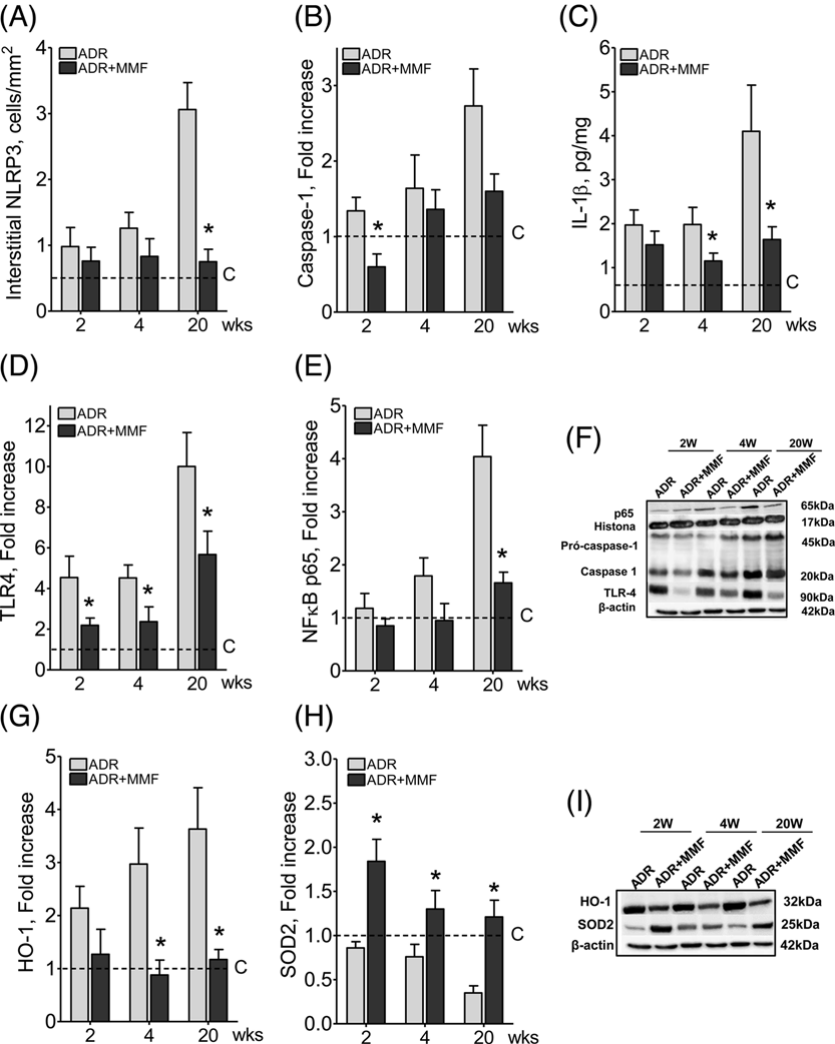
Effect of MMF treatment on innate immunity after adriamycin injection Effect of MMF treatment on interstitial abundance of NLRP3 (**A**), Caspase-1 (**B**), IL-1β (**C**), TLR4 (**D**), and nuclear NF-κB (**E**), with representative Western blot images (**F**). The renal content of HO-1 (**G**) and SOD2 (**H**), along with representative Western blot images (**I**) are also shown, *n*=10 per group. Student’s ‘*t*‘ test; *P<0.05 compared with ADR.

**Figure 10:**
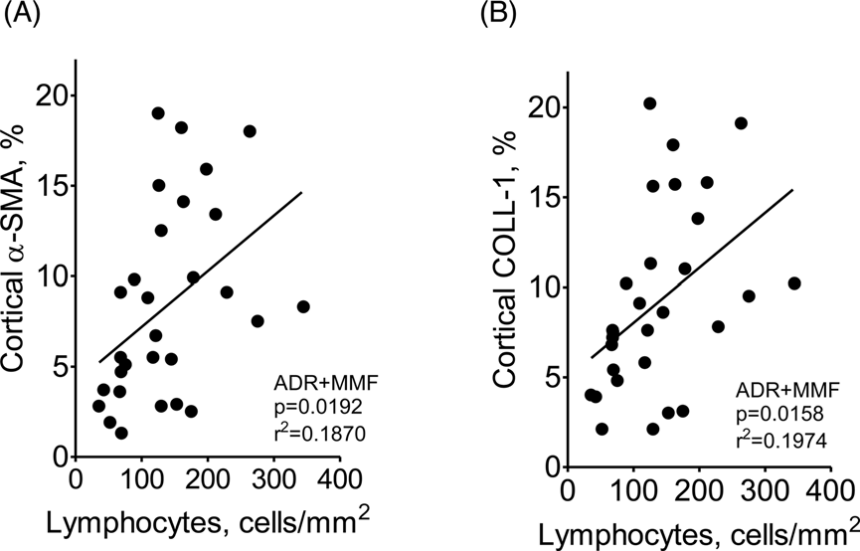
Linear correlation (Pearson’s correlation coefficient) between the intensity of T-lymphocyte (CD3-positive) infiltration and the percent cortical area staining for α-SMA (A) or collagen-1 (B)

Figure 8 shows that MMF treatment attenuated lymphocyte infiltration and prevented the progression of albuminuria, α-SMA infiltration, and collagen-1 deposition, while glomerulosclerosis was attenuated at the early stages of the nephropathy. Treatment with MMF also diminished the renal content of NLRP3, Caspase-1, IL-1β, and TLR4, as well as the nuclear translocation of NF-κB (Figure 9). The renal abundance of HO-1 was decreased, while that of SOD2 was increased, in rats receiving MMF, suggesting strong attenuation of oxidative stress (Figure 9). Moreover, the density of renal lymphocyte infiltration correlated with the cortical content of α-SMA and collagen-1 (Figure 10).

**Figure 11:**
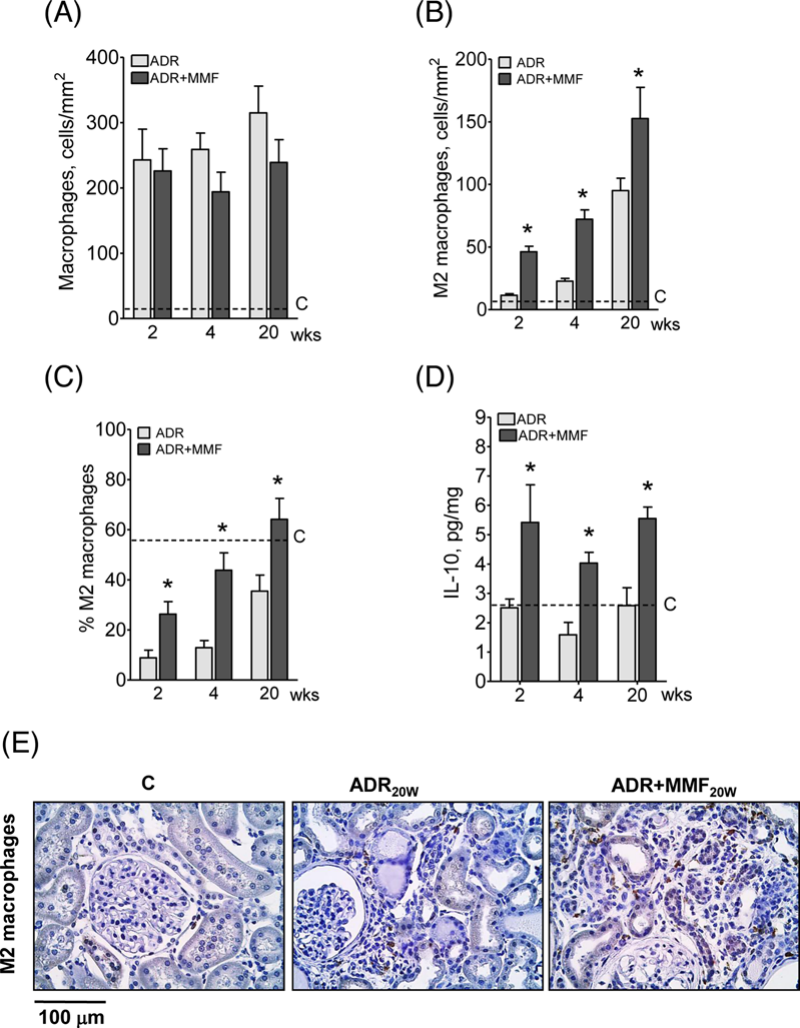
Effect of MMF treatment on polarization machophages and content of IL-10 after adriamycin injection Renal density of macrophages ED-1 (**A**), M2 macrophages (CD206+ cells) (**B**), percent M2 macrophages (**C**), and renal IL-10 abundance (**D**) 2, 4, and 20 weeks after ADR injection. Figure 11E shows representative microphotographs of M2 macrophage detection by immunohistochemistry in C and 20 weeks after ADR injection in untreated and MMF-treated rats, *n*=10 per group. Student’s ‘*t*’ test; *P<0.05 compared with ADR.

Macrophage ED-1 infiltration density was not significantly altered by MMF (Figure 11A). However, the proportion of M2 macrophages was markedly increased by MMF (Figure 11B,C), possibly as a consequence of the increased abundance of IL-10 (Figure 11D).

## Discussion

As expected [37], rats treated with ADR developed massive albuminuria, hypoalbuminemia, weight loss, anemia, and hyperlipidemia. These changes were accompanied by renal hypertrophy, hypertension, glomerulosclerosis, and interstitial inflammation/fibrosis, as well as by renal functional loss 20 weeks after ADR injection, an unusually long observation time for this model.

Our finding of increased NGAL excretion in the urine 1 week after ADR, when SCr was not yet elevated, indicates the early occurrence of tubular injury. Of note, NGAL has recently been described as a biomarker that may become useful for the early detection of CKD [38]. Also indicative of early tubular damage in ADR rats was the finding of elevated serum K^+^ at the same time.

Proteinuria, a hallmark of the ADR model, is a sign of glomerular damage and a predictor of progression of human renal disease. Several studies have shown a positive correlation between the degree of proteinuria and the progression of the disease, suggesting that the presence of protein in the tubular fluid may be noxious to tubular cells [6,7,39–41]. These findings do not prove causality. However, several *in vitro* studies [42–45] have provided strong evidence that proteinuria is indeed toxic to tubular cells.

Tubular damage was not the only consequence of ADR-induced proteinuria. ADR administration was also associated with early interstitial infiltration by lymphocytes and macrophages, as well as overexpression of MCP-1, consistent with the concept that exposure of tubular cells to excess protein leads to phenotypic changes conducive to inflammation and fibrosis. Previous studies showed that exposure to albumin stimulates tubular cells to synthesize MCP1 and RANTES, recruiting leukocytes, with further release of cytokines, and promoting fibrosis through activation of protein kinase C, MAP kinase, and/or the NF-κB system [13,42–45]. Other studies have shown that exposure of PTCs to high concentrations of albumin enhances the expression of type II transforming growth factor receptor in cultured PTCs [46]. In line with these earlier observations, albuminuria in the present study correlated with the density of infiltration by myofibroblasts and with the intensity of collagen deposition.

Also consistent with previous studies [47–49], intense lymphocyte infiltration, indicating early activation of adaptive immunity, was observed at the renal interstitial area only 2 weeks after ADR injection. In addition, early activation of at least two major innate immunity pathways was observed in this group: the TLR4/NF-κB/IL-6 pathway and the NLRP3 inflammasome/Caspase-1 pathway, along with IL-1β, one of its most potent end products. Moreover, the urinary excretion of albumin correlated positively with the renal abundance of IL-1β. Together, these findings suggest that exposure to excess albumin may have triggered the activation of innate immunity in tubular cells.

Activation of innate immunity is one of the potential stimuli for renal inflammation in renal disease, and may be the link between exposure to excess protein and the development of a proinflammatory phenotype. Previous studies showed increased expression of IL-1β and caspase-1 in the tubular epithelium of proteinuric CKD patients [11,28]. Simultaneous activation of the NF-κB system and MCP-1-dependent interstitial inflammation was reported in two heavily proteinuric rat models, Nx and Heymann nephritis [23,50]. BSA was shown to induce the activation of the NLRP3 inflammasome in diabetic nephropathy [13]. Thus, abundant *in vivo* and *in vitro* evidence support the notion that exposure of PTC to excess albumin can activate innate immunity.

Consistent with previous observations and with our *in vivo* data, we showed in the present study that exposure of cultured murine PTCs to excess albumin augmented the production of NLRP3, Caspase-1, and IL-1β, indicating that the NLRP3 inflammasome pathway was activated. In addition, the increase in the renal content of TLR4 and IL-6 suggests that the NF-κB system was also set in motion. The marked and simultaneous increase in the production of MCP-1 indicates that activation of innate immunity in these cells was associated with the development of a proinflammatory phenotype. The concomitant increase in the production of α-actin and collagen is consistent with the concept of epithelium-to-mesenchymal transformation [51–53], leading PTCs to behave as myofibroblasts, thus promoting directly the development of renal fibrosis. Our observation that silencing the *TLR4* and *NLRP3* genes abrogated the albumin-stimulated production of inflammatory mediators and collagen indicates that activation of at least some innate immunity pathways may indeed constitute the link between excess intratubular protein and the development of a proinflammatory phenotype by PTC.

The mechanisms by which excess albumin activates innate immunity are unclear. Excess albumin is known to promote intense endocytic and proteolytic cell activity, and may activate innate immunity through the rupture of lysosomes and the consequent cell accumulation of hydrolysis products and membrane debris that may act as danger-associated molecular patterns (DAMPs) [8,54]. Additional contribution may have derived from renin–angiotensin activation and oxidative stress associated with intense protein endocytosis and digestion [53]. Oxidative stress may exert a proinflammatory action by several mechanisms, including activation of the NF-κB system [43,55]. Previous studies showed that albuminuria induces ROS production, associated with activation of both the NF-κB and NLRP3 pathways [56,57]. In the present study, we showed increased abundance of HO-1 in the ADR group, a well-known adaptation against oxidative stress and other cytotoxic effects. Consistent with these results, the renal content of SOD2, another protective molecule against reactive oxygen species [58], was decreased in the long run in ADR animals, suggesting deficient removal of ROS, which may have further contributed to oxidative stress.

We have recently demonstrated that innate and acquired immunity are activated in parallel in the Nx model [9]. Other studies provided a possible mechanistic explanation for this link by showing that NLRP3 is expressed in CD4 lymphocytes, acting as a transcription factor [59]. The present study provides clear evidence that adaptive immunity was involved in the renal injury and inflammation associated with ADR administration. Renal lymphocyte infiltration was seen as early as 2 weeks after ADR administration, and its density correlated with the abundance of NLRP3 and IL-1β. However, the most compelling evidence of the importance of early activation of adaptive immunity in this setting is the protective effect of MMF treatment, especially in advanced phases. MMF reduced lymphocyte infiltration in the early stages of ADR and prevented the progression of interstitial fibrosis, even though albuminuria remained markedly elevated. It is possible that part of this beneficial effect was due to inhibition of B cells, since rituximab, an anti-CD20 agent, was shown to protect podocytes and ameliorate proteinuria in ADR-treated rats [60].

One novel finding of this study was that treatment with MMF diminished the renal abundance of TLR4, NLRP3, Caspase-1 and IL-1β, as well as the nuclear translocation of NF-κB. Accordingly, the renal content of collagen-1 and the infiltration by myofibroblasts were also reduced. These findings underline the intense cross-talk between innate and adaptive immunity. The mechanisms by which inhibition of adaptive immunity by MMF so deeply influenced innate immunity are unclear. One theoretical possibility [61] is that since innate and adaptive immunity can stimulate each other – innate immunity activation recruits lymphocytes, whereas the resulting inflammation leads to the release of DAMPs from injured cells – interruption of this vicious cycle by MMF would diminish the activity of both.

MMF can influence innate immunity more directly. In *in vitro* studies, mycophenolic acid was shown to inhibit phosphorylation of NF-κB in PTCs exposed to oxidative stress [62] or albumin overload [63]. In the present study, the renal content of HO-1 was decreased, while that of SOD2 was increased, in MMF-treated rats, suggesting that MMF may have influenced innate immunity through mitigation of renal oxidative stress. An additional influence of MMF was the shifting of macrophages toward the M2 phenotype, in which the expression of NLRP3, caspase-1, and IL-1β is low [61,64], without reducing the macrophage infiltration density. Although some subpopulations of M2 macrophages may be profibrotic [61], the observed up-regulation of IL-10, an anti-inflammatory cytokine [61,64], strongly suggests that, in the present study, the M2 polarization did exert an antifibrotic effect.

In summary, both innate and adaptive immunity are activated and interact in the ADR model. Both are involved in the toxic effect of excess albumin on PTC, leading to inflammation and fibrosis. Inhibition of any of them, *in vitro* with RNA silencing or *in vivo* with MMF, can decrease these toxic effects. These findings may contribute to build innovative strategies to prevent the progression of CKD in proteinuric diseases.

## Clinical perspectives

- In the present study, we investigated the role of innate immunity in the pathogenesis of the nephropathy associated with ADR administration in rats.
- We were able to show that the renal inflammation associated with this experimental model is accompanied by activation of the NLRP3 inflammasome, and that the proinflammatory phenotype evoked by exposure of cultivated PTCs to high albumin concentrations is abrogated by silencing of the NLRP3 pathway.
- In addition, simultaneous administration of MMF lowered the renal lymphocyte infiltration and shifted infiltrating macrophages to the M2 phenotype, while preventing innate immunity activation and the development of renal injury and inflammation.
- We are convinced that the present study can contribute significantly to a better understanding of the pathogenesis of CKD associated with proteinuric conditions, and to the search for innovative strategies aimed at detaining CKD progression.

### Supporting information

**Fig. S1:** Albuminuria 24h (A), tail-cuff pressure (B), serum creatinine (C), weight/body weight (D), glomerulosclerosis % (E) and cortical Collagen-1 (F), 20 weeks after ADR administration. C n= 9, ADR_2w_ n=12, ADR_4w_ n=12, ADR_20w_ n=10. ANOVA ^a^p<0.05 vs. C; ^b^p<0.05 vs. ADR_2w_; ^c^p<0.05 vs. ADR_4w_

**Fig. S2:** Urinary excretion of neutrophil gelatinase-associated lipocalin (NGAL) (A) and serum K+ concentration (B) 2, 4 and 20 weeks after ADR injection. C n= 9, ADR2w n=12, ADR4w n=12, ADR20w n=10. ANOVA ^a^p<0.05 vs. C; ^b^p<0.05 vs. ADR_2w_; ^c^p<0.05 vs. ADR_4w_

**Fig. S3:** Interstitial infiltration by T lymphocytes (CD3-positive) (A), macrophages ED-1 (B) and monocyte chemoattractant protein 1 (MCP-1) (C), 2, 4 and 20 weeks after ADR injection. C n= 9, ADR2w n=12, ADR4w n=12, ADR20w n=10. ANOVA ^a^p<0.05 vs. C; ^b^p<0.05 vs. ADR_2w_; ^c^p<0.05 vs. ADR_4w_

**Fig. S4:** Representative microphotographs of glomerulosclerosis in PAS-stained kidney sections (A); interstitial collagen-1 deposition detected by immunohistochemistry (B); renal infiltration by myofibroblasts in sections stained by immunohistochemistry for α-SMA (C); interstitial infiltration by macrophages (D) and T lymphocytes (CD3-positive) (E) detected by immunohistochemistry 20 weeks after ADR injection.

**Fig. S5:** Left panel: correlation (Pearson’s correlation coefficient) between albuminuria and the percent area staining positively for α-smooth muscle actin (α-SMA) or collagen-1. Right panel: correlation between albuminuria and the percent area staining positively for collagen-1

**Fig. S6:** Left panel: correlation (Pearson’s correlation coefficient) between the density of renal infiltration by T lymphocytes (CD3-positive) and that of cells staining positively for NLRP3. Right panel: correlation between the density of renal infiltration by lymphocytes and the renal content of IL-1β

**Fig. S7:** Percent of viable cultivated cells, as assessed by the 3-(4,5-dimethylthiazol-2-yl)-2,5-diphenyltetrazolium (MTT) test, in cells exposed to high albumin concentrations and either scramble or silencing RNA for TLR-4 or NLRP-3. No significant difference was observed.

## Abbreviations

ADR: adriamycin nephropathy
CKD: chronic kidney disease
DAMP: danger-associated molecular pattern
HO-1: heme oxygenase-1
MMF: mycophenolate mofetil
Nx: 5/6 renal ablation
PTC: proximal tubular cell
SCr: serum creatinine
SOD2: superoxide dismutase 2
TG: triglyceride
TLR: toll-like receptor
