# Supplementary material for: Simultaneous activation of innate and adaptive immunity participates in the development of renal injury in a model of heavy proteinuria
Source: Biosci Rep. 2018 Jul 13;38(4):BSR20180762. doi: 10.1042/BSR20180762 (PMC6043717; doi:10.1042/BSR20180762)
Supplement: Supplementary file 1 [file bsr20180762_Supp1.pdf]

Figure S1

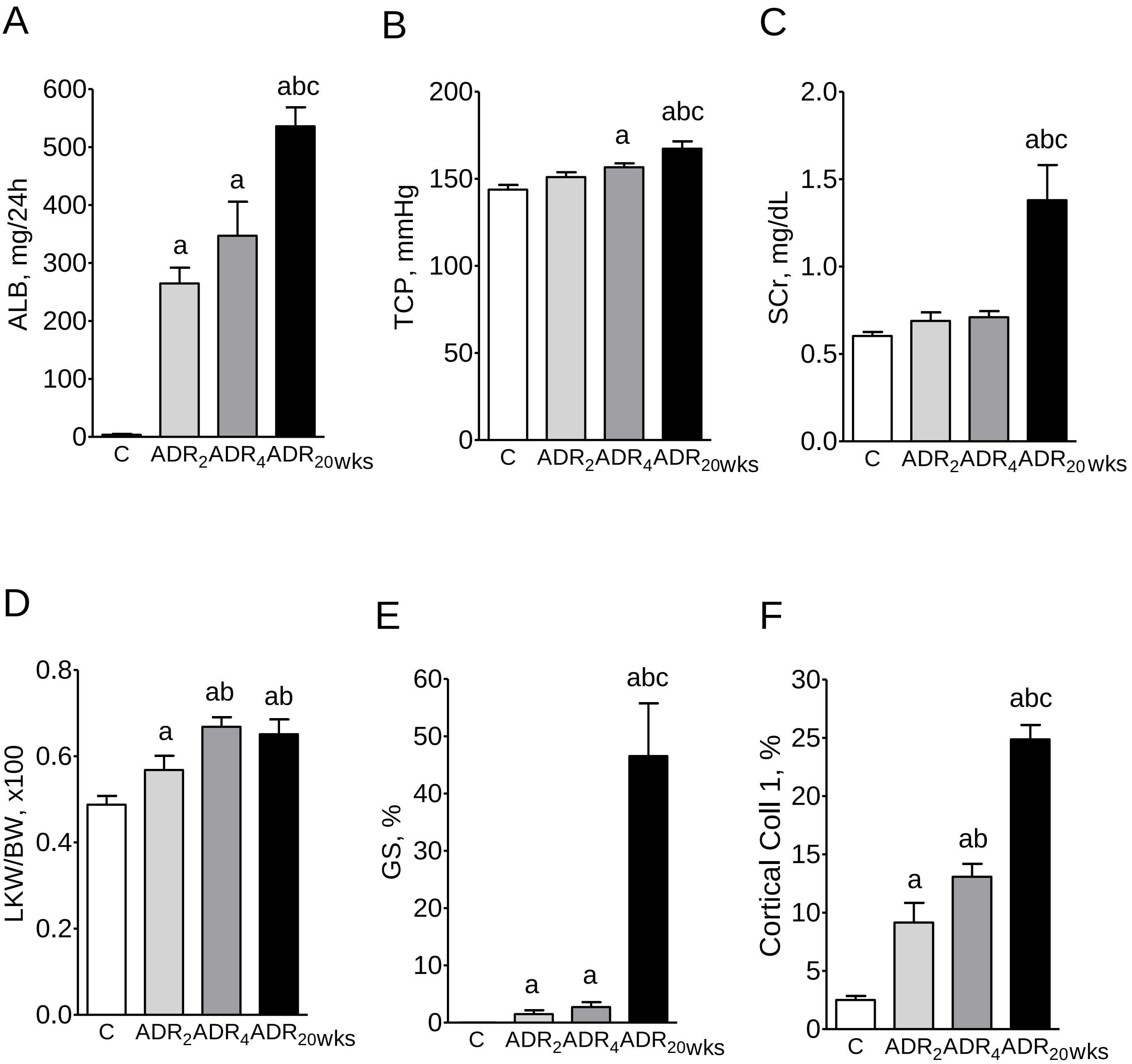

Fig. S1 - Albuminuria 24h (A), tail-cuff pressure (B), serum creatinine (C), weight/body weight (D), glomerulosclerosis % (E) and cortical Collagen-1 (F), 20 weeks after ADR administration. C n= 9, ADR_{2w} n=12, ADR_{4w} n=12, ADR_{20w} n=10. ANOVA $^{a}p<0.05$ vs. C; $^{b}p<0.05$ vs. ADR_{2w}; $^{c}p<0.05$ vs. ADR_{4w}

Figure S2

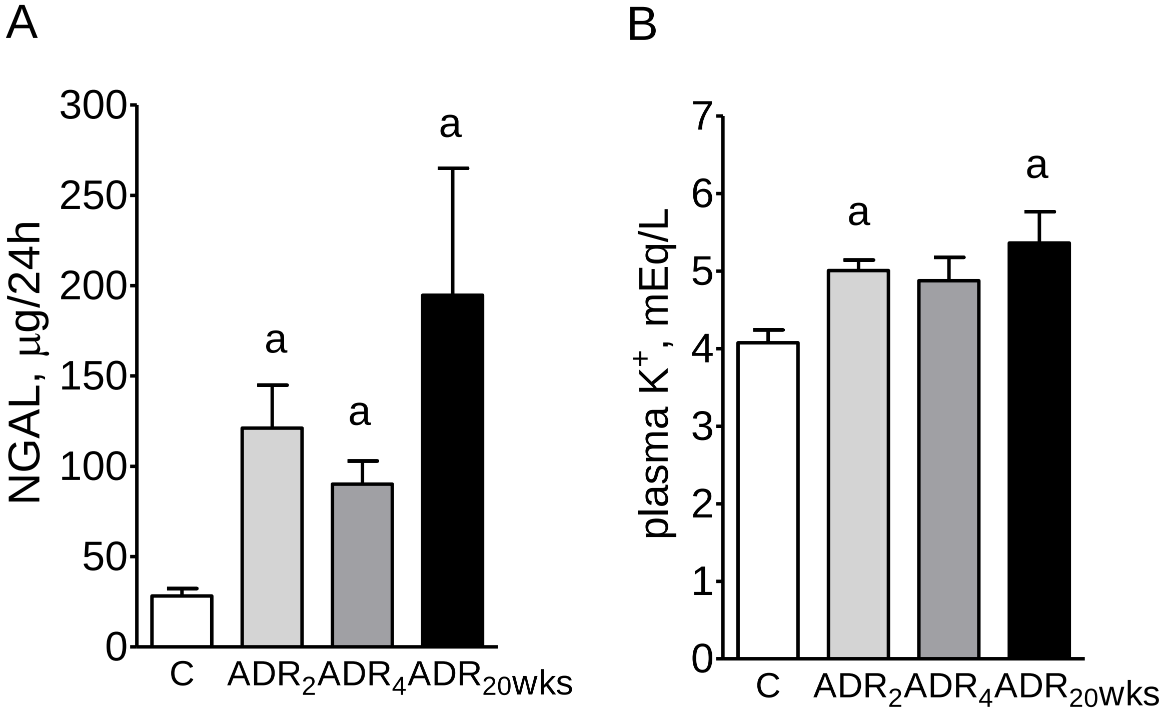

Fig. S2 - Urinary excretion of neutrophil gelatinase-associated lipocalin (NGAL) (A) and serum K+ concentration (B) 2, 4 and 20 weeks after ADR injection. C n= 9, ADR2w n=12, ADR4w n=12, ADR20w n=10. ANOVA $^{a}p<0.05$ vs. C; $^{b}p<0.05$ vs. ADR_{2w}; $^{c}p<0.05$ vs. ADR_{4w}

Figure S3

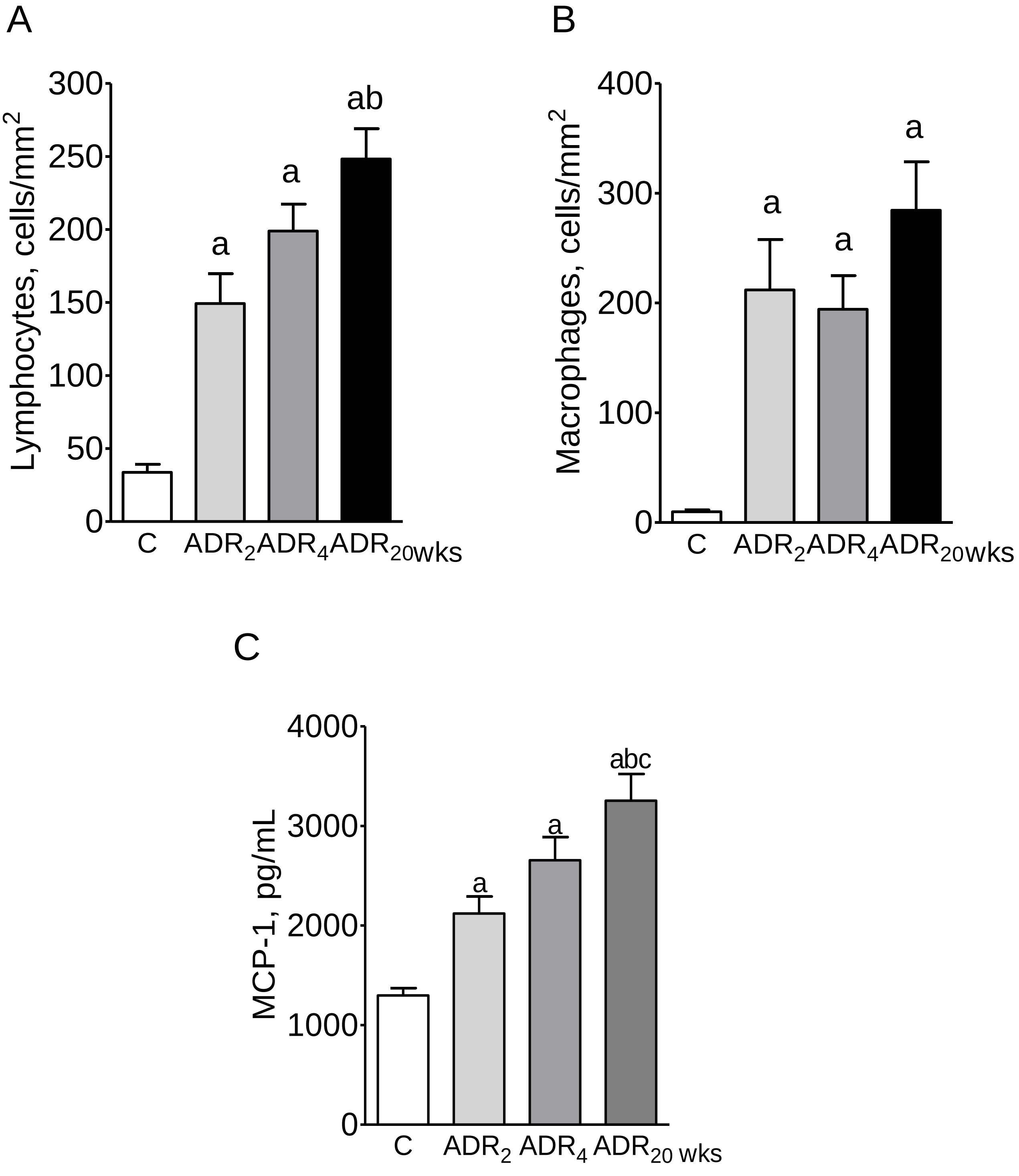

Fig.S3 - Interstitial infiltration by T lymphocytes (CD3-positive) (A), macrophages ED-1 (B) and monocyte chemoattractant protein 1 (MCP-1) (C), 2, 4 and 20 weeks after ADR injection. C n= 9, ADR2w n=12, ADR4w n=12, ADR20w n=10. ANOVA $^{a}p<0.05$ vs. C; $^{b}p<0.05$ vs. ADR$_{2w}$; $^{c}p<0.05$ vs. ADR$_{4w}$

Figure S4

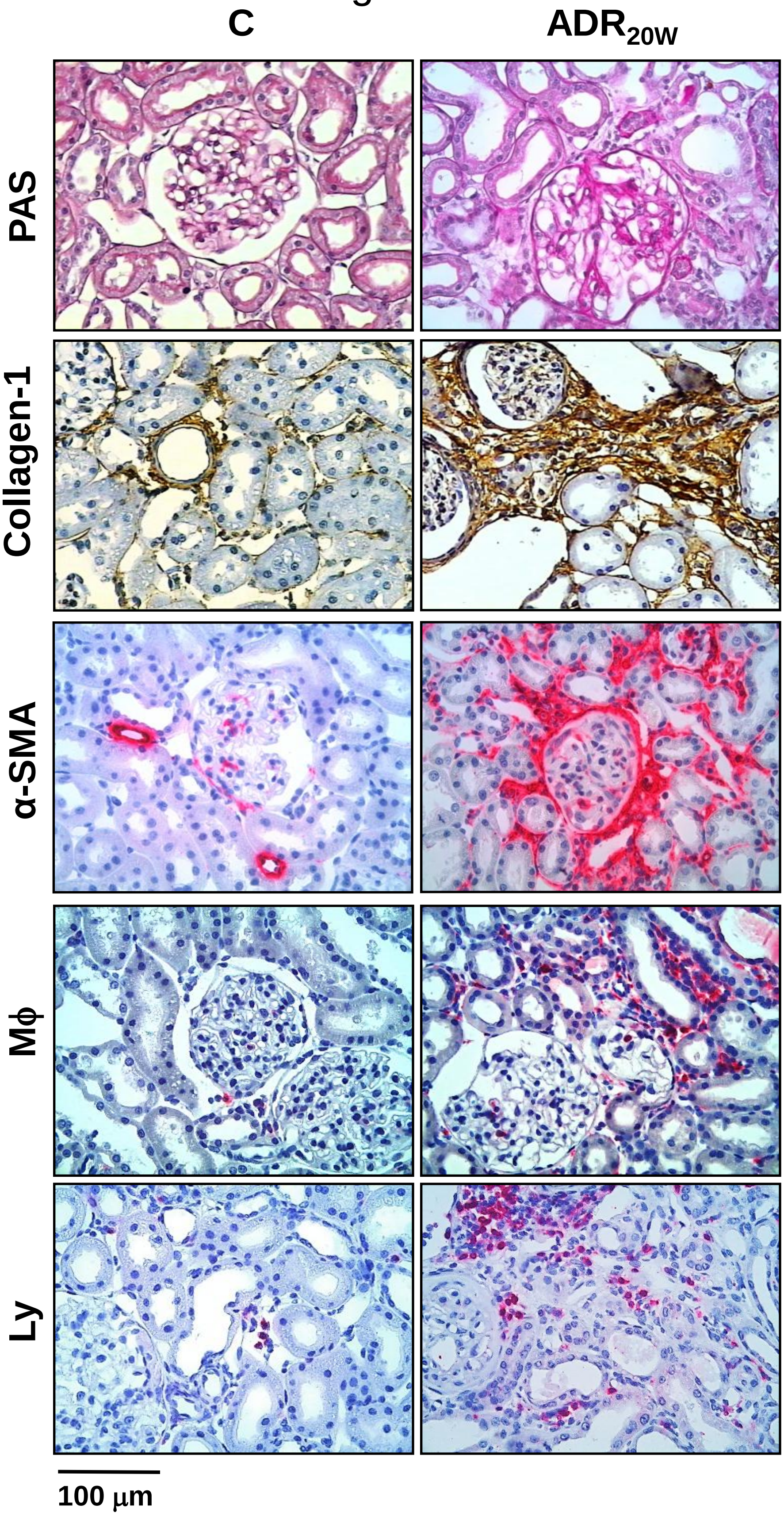

Fig. S4 - Representative microphotographs of glomerulosclerosis in PAS-stained kidney sections (A); interstitial collagen-1 deposition detected by immunohistochemistry (B); renal infiltration by myofibroblasts in sections stained by immunohistochemistry for α-SMA (C); interstitial infiltration by macrophages (D) and T lymphocytes (CD3-positive) (E) detected by immunohistochemistry 20 weeks after ADR injection.

Figure S5

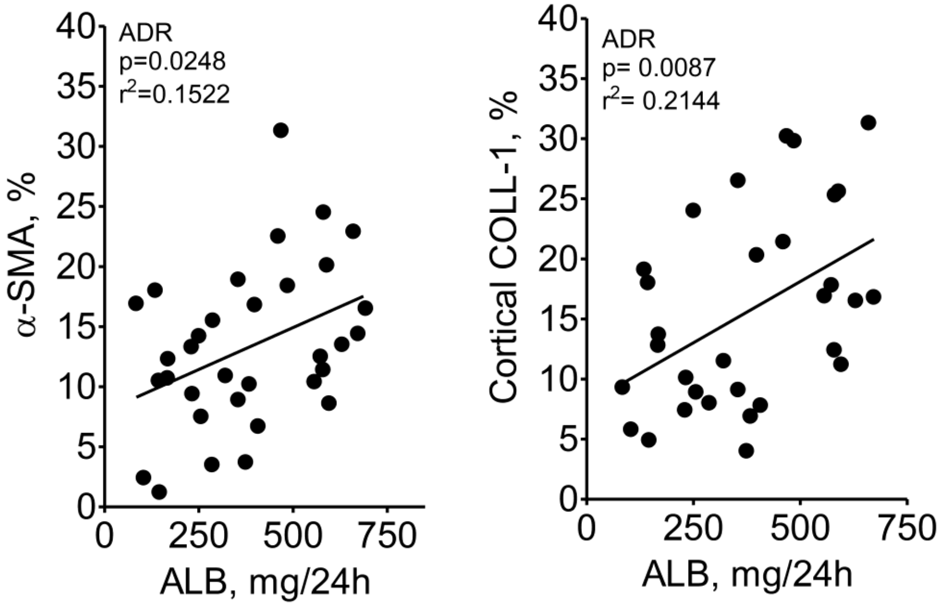

Fig. S5 – Left panel: correlation (Pearson's correlation coefficient) between albuminuria and the percent area staining positively for α-smooth muscle actin (α-SMA) or collagen-1. Right panel: correlation between albuminuria and the percent area staining positively for collagen-1

Figure S6

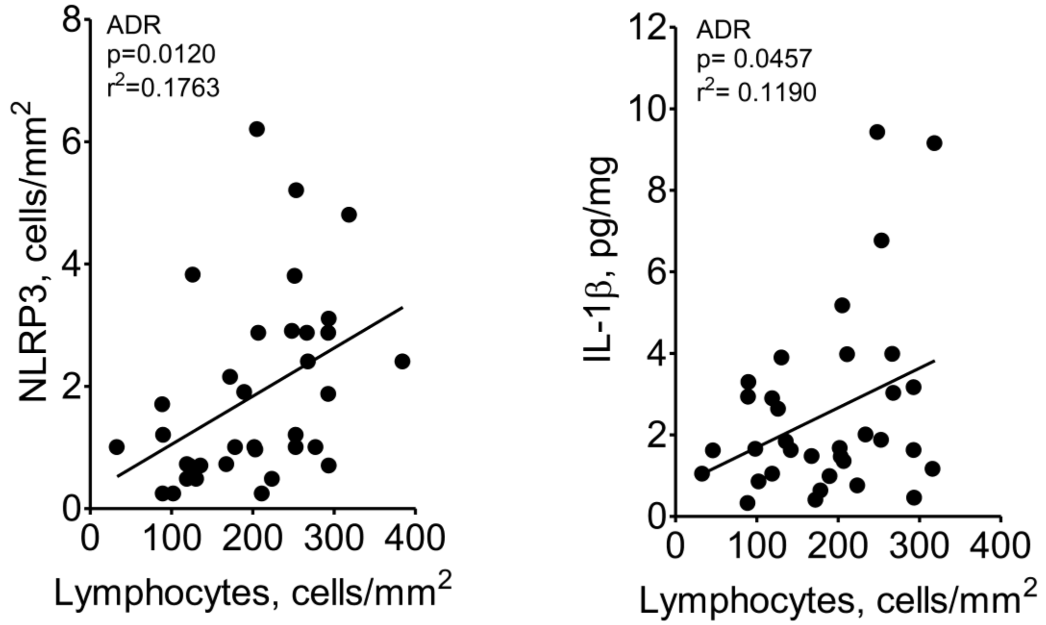

Fig. S6 – Left panel: correlation (Pearson's correlation coefficient) between the density of renal infiltration by T lymphocytes (CD3-positive) and that of cells staining positively for NLRP3. Right panel: correlation between the density of renal infiltration by lymphocytes and the renal content of IL-1β

Figure S7

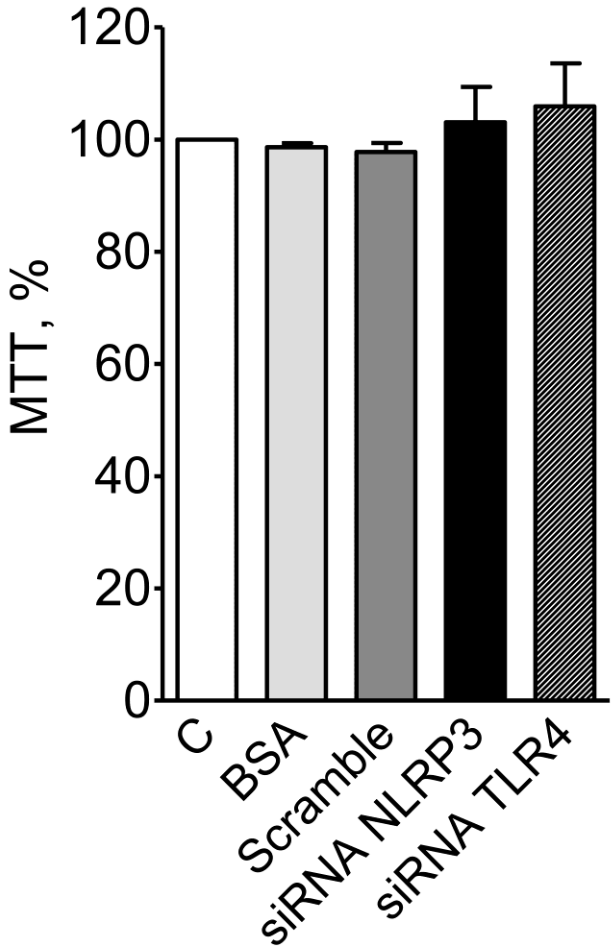

Fig. S7 – Percent of viable cultivated cells, as assessed by the 3-(4,5-dimethylthiazol-2-yl)-2,5-diphenyltetrazolium (MTT) test, in cells exposed to high albumin concentrations and either scramble or silencing RNA for TLR-4 or NLRP-3. No significant difference was observed.
